# Supplementary material for: Identification of Sample Processing Errors in Microbiome Studies Using Host Genetic Profiles
Source: bioRxiv. 2025 Sep 12:2025.09.07.674724. Preprint. [Version 1] doi: 10.1101/2025.09.07.674724 (PMC12440029; doi:10.1101/2025.09.07.674724)
Supplement: Supplement 1 [file NIHPP2025.09.07.674724v1-supplement-1.pdf]

bioRxiv preprint doi: https://doi.org/10.1101/2025.09.07.674724; this version posted September 12, 2025. The copyright holder for this preprint (which was not certified by peer review) is the author/funder, who has granted bioRxiv a license to display the preprint in perpetuity. It is made available under aCC-BY 4.0 International license.

Supplementary Material

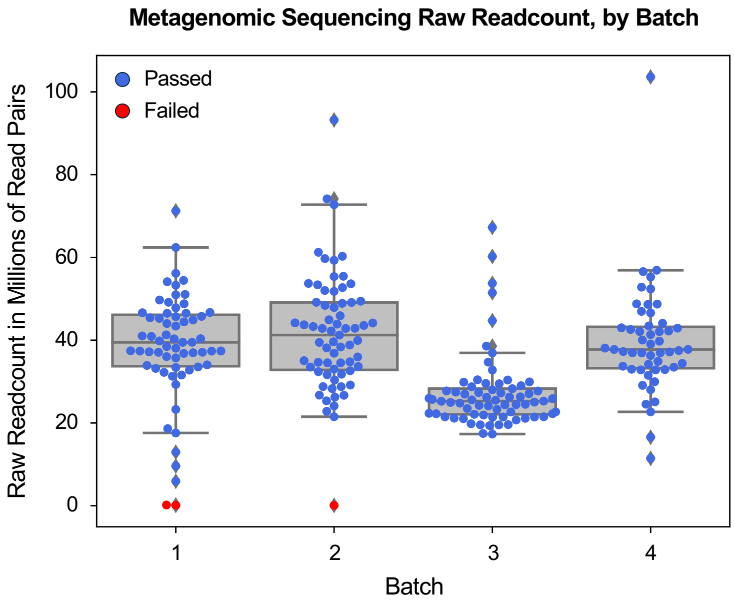

Supplementary Figure 1 | Raw readcount of metagenomic samples, by batch. Three samples failed metagenomic sequencing with raw readcounts < 1M read pairs (91,462, 109,247, and 178,358 read pairs; samples marked with red points) and were thus removed prior to downstream analyses.

bioRxiv preprint doi: https://doi.org/10.1101/2025.09.07.674724; this version posted September 12, 2025. The copyright holder for this preprint (which was not certified by peer review) is the author/funder, who has granted bioRxiv a license to display the preprint in perpetuity. It is made available under aCC-BY 4.0 International license.

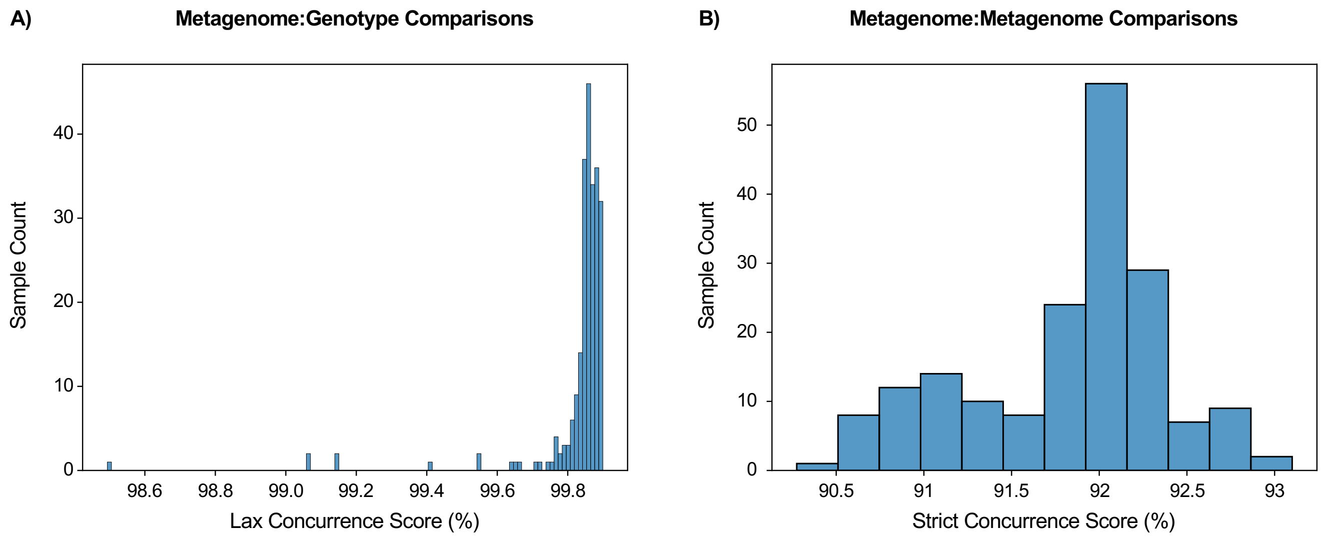

Supplementary Figure 2 | Score distributions for comparisons that are not indicative of sample processing error. (A) Lax concurrence score distribution for matched metagenome:genotype comparisons (comparisons of metagenomes with their donors by label) with concurrence >98%, our operational threshold for determining true donor identity. **(B)** Strict concurrence score distribution for metagenome:metagenome comparisons between samples from the same donor with concurrence >90%, our threshold for identifying samples from the same donor.

bioRxiv preprint doi: https://doi.org/10.1101/2025.09.07.674724; this version posted September 12, 2025. The copyright holder for this preprint (which was not certified by peer review) is the author/funder, who has granted bioRxiv a license to display the preprint in perpetuity. It is made available under aCC-BY 4.0 International license.

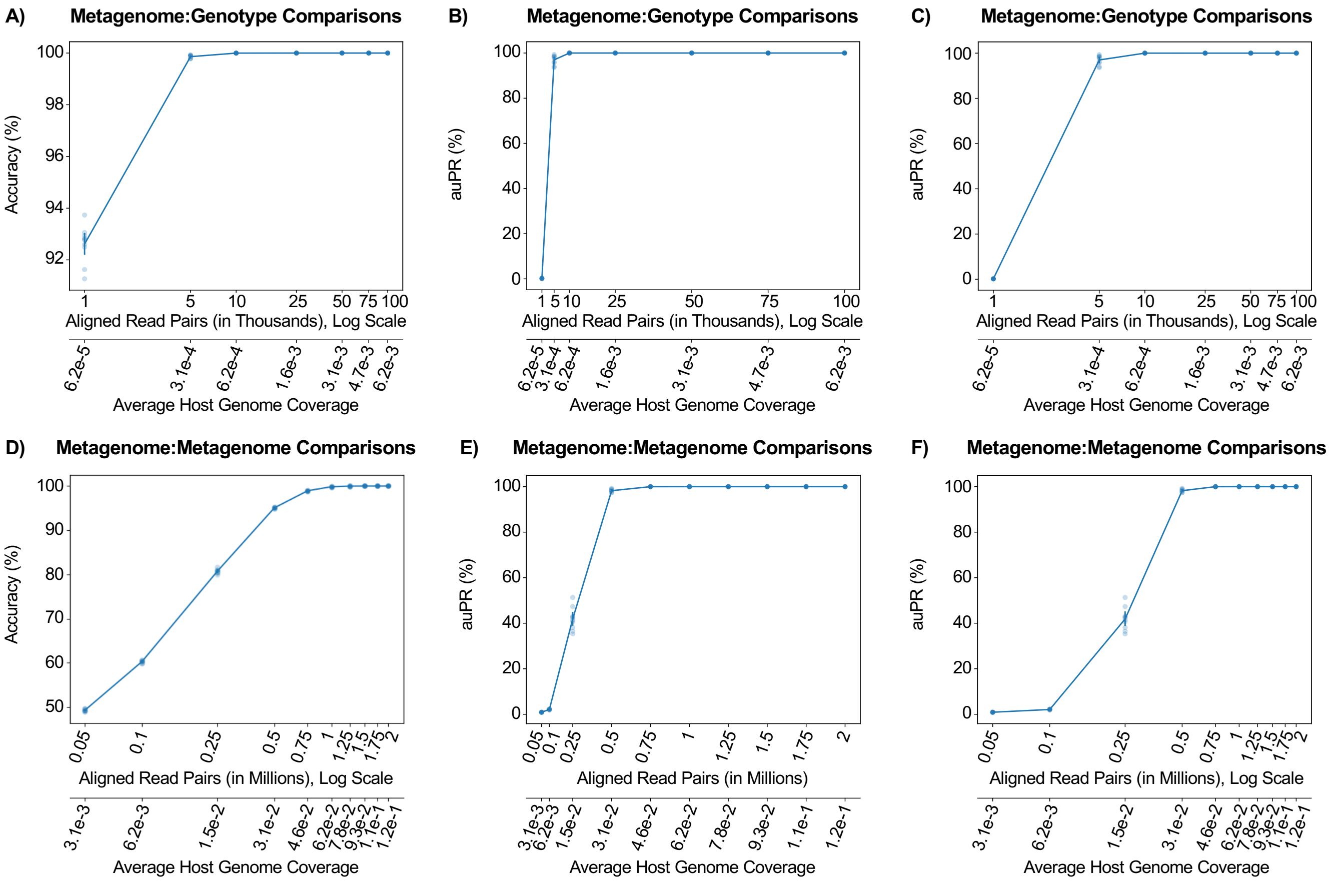

Supplementary Figure 3 | Concurrence analysis is robust for low sequencing coverage. Same as **Fig. 4**, but with read count displayed in log-scale to better show accuracy at lower read thresholds, as well as area under the precision-recall curve (auPR) instead of accuracy.
